# Leveraging the metacoupling framework for sustainability science and global sustainable development

**DOI:** 10.1093/nsr/nwad090

**Published:** 2023-03-31

**Authors:** Jianguo Liu

**Affiliations:** Center for Systems Integration and Sustainability, Department of Fisheries and Wildlife, Michigan State University, East Lansing, MI 48823, USA

**Keywords:** biodiversity, ecosystem services, human–nature interactions, planetary boundaries, telecoupling, sustainable development

## Abstract

Sustainability science seeks to understand human–nature interactions behind sustainability challenges, but has largely been place-based. Traditional sustainability efforts often solved problems in one place at the cost of other places, compromising global sustainability. The metacoupling framework offers a conceptual foundation and a holistic approach to integrating human–nature interactions within a place, as well as between adjacent places and between distant places worldwide. Its applications show broad utilities for advancing sustainability science with profound implications for global sustainable development. They have revealed effects of metacoupling on the performance, synergies, and trade-offs of United Nations Sustainable Development Goals (SDGs) across borders and across local to global scales; untangled complex interactions; identified new network attributes; unveiled spatio-temporal dynamics and effects of metacoupling; uncovered invisible feedbacks across metacoupled systems; expanded the nexus approach; detected and integrated hidden phenomena and overlooked issues; re-examined theories such as Tobler's First Law of Geography; and unfolded transformations among noncoupling, coupling, decoupling, and recoupling. Results from the applications are also helpful to achieve SDGs across space, amplify benefits of ecosystem restoration across boundaries and across scales, augment transboundary management, broaden spatial planning, boost supply chains, empower small agents in the large world, and shift from place-based to flow-based governance. Key topics for future research include cascading effects of an event in one place on other places both nearby and far away. Operationalizing the framework can benefit from further tracing flows across scales and space, uplifting the rigor of causal attribution, enlarging toolboxes, and elevating financial and human resources. Unleashing the full potential of the framework will generate more important scientific discoveries and more effective solutions for global justice and sustainable development.

## Teaser

This article highlights major advances in scientific discoveries for sustainability science from applying the integrated metacoupling framework, illustrates implications for global sustainable development, and offers future perspectives to further empower the framework.

## INTRODUCTION

The world is facing numerous sustainability challenges [1–3]. They include air pollution, biodiversity loss, climate change, deterioration of ecosystem services, disasters, disease spread, energy crises, food insecurity, land degradation, ocean acidification, overpopulation, poverty, species invasion, war, and water shortages and pollution.

Sustainability challenges are largely outcomes of complex human–nature interactions. Sustainability science aims to understand the complexity of human–nature interactions or society–nature interactions [4], and has developed rapidly in the past two decades [5]. While sustainability science has generated many useful insights, the focus has often been on specific places separately. However, a challenge such as COVID-19 originating in one place affects not only that place but also many other places and even the rest of the world. Thus, a new field—spatial sustainability science—is emerging to promote studies on spatial dynamics and human–nature interactions across space worldwide for global sustainable development [6].

In 2015, to solve global sustainability challenges, 193 countries adopted the United Nations’ 17 Sustainable Development Goals (SDGs) [7]. The United Nations (UN) seeks to achieve these goals around the world by 2030, such as to ‘end poverty in all forms everywhere’ (SDG 1). However, efforts for achieving SDGs in one place could affect progress in other places positively (synergies) or negatively (trade-offs) [8–10]. Furthermore, synergies and trade-offs change over time and at different economic development levels [11]. Thus, there is a strong need to have a framework that can help assess human–nature interactions, as well as SDG synergies and trade-offs, within and among adjacent and distant places, and help discover and manage hidden phenomena and complex feedbacks that may not be apparent when focusing on a particular place [12].

In 2017, an integrated framework of metacoupling (human–nature interactions within a system, as well as between adjacent systems and between distant systems) [13] was published to meet the need as mentioned above. It has been applied to advance sustainability science and to understand and solve global sustainability challenges. The applications have spread across various places, sectors, and issues worldwide. They range from the Arctic to temperate to tropical to Antarctic regions [14–16], from terrestrial systems to aquatic systems (marine, freshwater, coastal) [14–16,17], from rural to urban areas [18,19], and from upstream to midstream and downstream [20]. They are used in different sectors, such as agriculture, fisheries, and tourism [16–21]. They also address a wide range of issues, such as those related to planetary boundaries (e.g. pollution, biodiversity, biogeochemical flows, climate change, freshwater use, land use) [22,23], foreign investment [22], impacts of international trade on SDGs (e.g. [8]) and deforestation [24], benefits of food imports to food security and biodiversity conservation in countries with biodiversity hotspots [25], and the food–energy–water–CO_2_ nexus [26].

To further empower the framework, this article first provides an overview of the framework, then highlights major advances in scientific discoveries and illustrates implications for global sustainability based on the existing applications of the framework (Table 1), and finally offers future perspectives to fill important knowledge gaps and needs for tools and policy innovations. The scientific discoveries are helpful for fostering sustainability science (especially spatial sustainability science). The implications are relevant for more effective governance, management, spatial planning, and ecosystem restoration toward sustainability.

**Table 1.** Example functions of the metacoupling framework.

Functions	Illustrative studies
***Major advances in scientific discoveries***	
Revealing effects of metacoupling on SDG performance and spatial interactions	• Metacoupling has important impacts on progress toward SDGs across borders. SDG targets had the highest scores under telecoupling (trade among distant countries), followed by pericoupling (trade among adjacent countries) and intracoupling (no trade) [8]. Developed countries benefitted more from telecoupling than pericoupling while developing countries suffered more from telecoupling than pericoupling [8].• Tourism and wildlife translocations led to synergies and trade-offs among SDGs within focal systems (sending or receiving systems) and across systems including spillover systems [9,10].
Untangling complex interactions among intracoupling, pericoupling, and telecoupling	• Intracoupling, pericoupling, and telecoupling have synergistic effects [66].• Pericoupling and telecoupling amplify intracoupling [67].• Increases in one type of coupling reduce other types of coupling [16] and one type of coupling generates benefits on one scale at the cost of other scales [16].• Telecoupling, pericoupling, and intracoupling interact indirectly [18,20].• Multiple types of metacoupling interact positively or negatively [68,69].
Unveiling spatiotemporal dynamics and effects of intracoupling, pericoupling, and telecoupling	• Intracoupling, pericoupling, and telecoupling change in the same or different directions over time and across space [70].• Effects of metacoupling differ in sending, receiving, and spillover systems [15].• Different types of resources (e.g. energy and water) are gained or lost across metacoupled systems [69].
Identifying new network attributes	• Metacoupled systems can be viewed as expanded versions of networks and have important network attributes such as distinct cliques and influential players [76–79].
Uncovering feedbacks across metacoupled systems	• Feedbacks (positive or negative) are common in metacoupled systems (e.g. through global soybean trade, payments for ecosystem services) although they take time to emerge [76,80–82].
Detecting and integrating hidden phenomena and overlooked issues	• Countries like Russia providing fertilizers for soybean production in Brazil are spillover countries of soybean production for exports to China and some European countries [56].• Spillover effects can be much larger than effects between sending and receiving systems [84].• There are many hidden stakeholders and unknown concerns for groundwater governance [66].• Water use, income of rural households, and effectiveness of afforestation are affected by local and nonlocal factors [85–87].• Major components related to ecosystem flows are integrated [75,89,90].
Expanding the nexus approach	• The metacoupling perspective expanded the traditional nexus approach from focus on connections among sectors within a place and at a particular scale to all sectors within as well as in adjacent and distant places and across multiple scales [91], with empirical testing of the energy–water nexus [69], food–energy–water–CO_2_ nexus [26], and virtual CO_2_–energy–land–water–nitrogen–financial capital nexus [68].
Re-examining theories	• Results from many metacoupling analyses invalidate Tobler's First Law of Geography [18,22,68,69,76,92], show a broader applicability of the metacoupling framework than the First Law [93], and point to the need to re-examine other relevant theories [57].
Unfolding transformation among noncoupling, coupling, decoupling, recoupling	• Metacoupling may experience four stages of transformation: noncoupling, coupling, decoupling, and recoupling [16,94].
***Implications for promoting global sustainability***	
Achieving SDGs across space worldwide	• The framework helps realize SDGs in a specific place as well as adjacent and distant places [12].
Amplifying benefits of ecosystem restoration across boundaries and across scales	• The metacoupling framework helps motivate ecosystem restoration to reduce trade-offs and enhance synergies among multiple SDGs at multiple spatial scales [97].• The beneficial effects on SDGs at the restoration place should minimize the negative effects on the SDGs in places both nearby and far away worldwide [97]• The framework is conducive to generate comprehensive information on the effects of ecosystem restoration in the restoration place, adjacent places, and distant places [98].
Augmenting transboundary management	• The framework is effective to guide transboundary management such as management of transboundary watersheds [99,100].
Broadening spatial planning	• Results from metacoupling studies can assist spatial planning at different scales to evade negative effects and enhance positive effects on sustainability [16].• Information from metacoupling research can promote spatial equity and justice [101].
Boosting supply chains	• A metacoupling-based network analysis offers a systematic approach to understand emerging food supply chains and boost robust community-supported fishery management under normal logistical and financial challenges and extraordinary situations due to crises such as COVID-19 and other emergencies [77].• A metacoupling lens is critical to bridge knowledge gaps generated by separate assessments of human or natural dynamics within individual fisheries [77].• A new seafood delivery program minimized COVID-19 transmission while increasing the number of customers, diversified and dispersed the demand-side seafood distribution, and enhanced network resilience [77].
Empowering small agents in the large world	• Increasing agency (capability of influencing the formation or operation of flows) enables small agents (e.g. smallholder farmers) to improve well-being and achieve SDGs [102].
Shifting from place-based to flow-based governance	• Shifts in the governance from focus on specific places independently (place-based) to flows among places nearby and far away (flow-based) can enhance global sustainability and improve spatial justice and equity [91,103].

* For information about the differences between the metacoupling approach vs. other alternatives, please see the text and the references cited in the table. Space limitation does not allow such comparisons in the table.

## OVERVIEW OF THE METACOUPLING FRAMEWORK

### Metacoupling concept and composition

**Figure 1.**
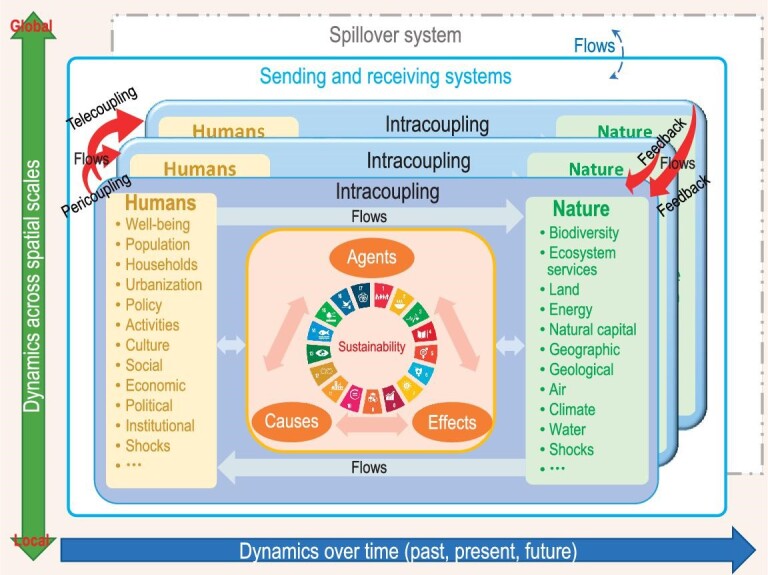
A diagram of the metacoupling framework and its relationship to sustainability. Each box indicates a coupled human and natural system, which consists of humans (e.g. populations, households) and nature (e.g. biodiversity, climate) that are connected by various flows (movements of information, people, organisms, energy, matter, products, capital, etc) and generate human–nature interactions within the system (intracoupling). Different systems are also connected by flows that lead to pericoupling (human–nature interactions between adjacent systems) and telecoupling (human–nature interactions between distant systems). Each system also includes causes (reasons behind the flows), agents (decision-making entities that facilitate the flows), and effects (e.g. ecological and socioeconomic consequences of the flows). The sending and receiving systems are represented by boxes with solid boundaries while the spillover system is represented by a box with dashed boundary lines. Metacoupling and other factors affect sustainability in each system and globally (represented by 17 UN Sustainable Development Goals (SDGs)). Human–nature interactions occur horizontally (among systems of different hierarchical structures at the same spatial scale), diagonally (among systems of different hierarchical structures at different spatial scales), and vertically (among systems of the same hierarchical structure across different spatial scales); and change over time. Credit (SDG symbols): [7] and [143].

Metacoupling encompasses human–nature interactions within a particular system (intracoupling), between adjacent systems (pericoupling), and between distant systems (telecoupling) [13] (Fig. 1). A system means a coupled human and natural system [27], such as a social–ecological system and a human–environmental system. It could be a place, including country, state/province, city, county, village, and watershed. There are many types of intracoupling in a specific system, such as farming, fishing, and timber harvesting. Pericoupling and telecoupling include trade, migration, species invasion, foreign investment, technology transfer, knowledge transfer, and tourism between adjacent systems and between distant systems, respectively.

Intracoupling, pericoupling, and telecoupling are umbrella concepts that expand, integrate, and compare disciplinary concepts. For example, traditional research on fishing has usually focused on fish products and socioeconomic benefits, although it has a lot of environmental consequences. Treating fishing in a specific place as intracoupling can expand the traditional focus of fishing by simultaneously integrating socioeconomic and environmental dimensions. Long-distance animal migration has been extensively studied by ecologists, with a focus on ecology and behavior [28]. However, animal migration also has important socioeconomic impacts [29]. If taking both ecological and socioeconomic dimensions into account, animal migration can be expanded as a telecoupling. In contrast, human migration has been extensively studied by social scientists, with a focus on socioeconomic aspects [30]. However, human migration has enormous environmental implications [30]. Considering both socioeconomic and environmental dimensions, human migration can be treated as a telecoupling. Viewing both animal migration and human migration under the telecoupling lens could also promote comparisons between these two processes often studied and managed separately.

An umbrella concept encompasses multiple concepts but does not replace them in specific contexts. For example, the concept of ecosystem services includes all kinds of ecosystem benefits to humans such as pollination and soil retention, but pollination and soil retention are still used when specifying the services of transferring pollen for fertilization and retaining soil, respectively [31]. Similarly, intracoupling consists of concepts such as farming and fishing within a place but does not constrain the use of farming or fishing when referring to growing crops on a farm or catching fish in a lake.

Metacoupling shapes global sustainability and has been gaining broad international interest. For example, telecoupling (a major aspect of metacoupling) is highlighted in many authoritative documents such as the Global Assessment Report on Biodiversity and Ecosystem Services [32]. It has been featured by the 2021 Nobel Prize Summit [33]. Telecoupling has also been used beyond academic research. It is the topic in the writing of high-level United Nations officials, entitled ‘Tele-coupling and why your choice matters for the planet’ [34]. In other words, telecoupling is relevant for everyone on Earth for global sustainability, as everything people produce, purchase, and consume influences global sustainability. Furthermore, metacoupling has been suggested as a key area of research for sustainability [35].

### Basic structure of the metacoupling framework

The metacoupling framework is the conceptual foundation to address all kinds of metacoupling. It is a combination of frameworks for intracoupling, pericoupling, and telecoupling [13] (Fig. 1). The telecoupling framework contains five interrelated components (systems, flows, agents, causes, and effects) [13,36]. Systems refer to coupled human and natural systems that are connected and form feedbacks through various flows (e.g. movements of capital, energy, information, matter, organisms, and people). Depending on the flow direction, systems can be classified as sending systems (that send flows out), receiving systems (that receive flows), and spillover systems (that are affected by the flows between sending and receiving systems) [13]. Agents refer to decision-making entities (e.g. animals, farmers, policy makers, traders) that facilitate various flows. Causes include reasons (e.g. cultural, ecological, geological, hydrological, political, socioeconomic factors) that generate various effects (e.g. biogeochemical, biological, ecological, hydrological, political, socioeconomic) [13]. The pericoupling framework is the same as the telecoupling framework except that sending and receiving systems are nearby rather than far away [13]. The intracoupling framework focuses on human–nature interactions within one system that consists of human and nature subsystems [13]. The interactions between human and nature subsystems are through flows and facilitated by agents within the focal system, are generated by different causes, and lead to different effects within both the focal system itself and the spillover system [13].

The metacoupling framework is applicable at multiple spatial and temporal scales (Fig. 1). There are three types of interactions including feedback between systems across space—horizontal, vertical, and diagonal. Horizontal interactions occur among systems at the same scale. For example, at the international scale, there are food imports and exports between adjacent countries and between distant countries in addition to food for domestic consumption. At the regional scale, there are flows of labor and materials between adjacent regions and between distant regions, in addition to labor and materials produced within each region. Vertical interactions occur among different scales (e.g. local, regional, and national) within the same hierarchical structure. Diagonal interactions occur across different scales among different systems. For example, illegal drugs from a region in Mexico are smuggled into and sold across the US [37]. While horizontal and vertical interactions have been widely studied (e.g. [5]), diagonal interactions have received relatively little attention in the sustainability literature. Furthermore, for each coupled human and natural system, there may be scale mismatches between human and natural components [38]. For example, differences in spatial scales of governance and spatial scales of ecological processes lead to spatial scale mismatches. Disturbances such as shocks within a system or interactions with other systems may generate effects with different spatial extents. The metacoupling framework designates spillover systems to accommodate the different spatial extents of the effects that go beyond the sending and receiving systems. To reflect metacoupling changes over time, the framework is also applicable at different temporal scales from the past to the present to the future (Fig. 1).

### Relationships between the metacoupling framework and other concepts, frameworks, and disciplines

The metacoupling framework builds on, expands, and integrates contributions of various concepts, frameworks, and disciplines. These include general systems theory [39], systems ecology [40], spatial ecology, landscape ecology, ecosystem ecology, spatial economics, geography, metapopulation [41], metacommunity [42], meta-ecosystems [43], scale [44], movements (e.g. flows of nutrients, animals) [45], teleconnection [46], globalization [47], world systems theory [48], Institutional Analysis and Development [49], spatial subsidy [50], spatial externalities [51], off-site effect [52], displacements [53], leakages and indirect land use changes [54], and ecosystem services [55]. If one uses the metaphors of wine and a wine bottle, the metacoupling framework is new wine in a new bottle (rather than ‘old wine in a new bottle’). Assuming one pours some old red wine and some old white wine into a new bottle, the mixed wine in the new bottle is new because it is neither red wine nor white wine anymore, although elements of red wine and white wine may remain. Similarly, after different types of coupling are placed under the metacoupling framework, they form interrelationships and emergent properties, although elements of different types of coupling remain. While each type of coupling may have been studied separately in the past, putting them together under a broader framework enables comparative studies (e.g. the relative importance of each coupling) and research on their total impacts and interactions simultaneously. Such integrated research can avoid biases, generate more complete information, enhance synergies, and reduce trade-offs among different types of coupling. This is similar to many other integrated and umbrella concepts such as ‘ecosystem services’, which encompass a variety of nature's benefits to humans (e.g. pollination, flood mitigation, soil retention, food provisioning), although each of these benefits had been studied separately for a long time.

There are a variety of differences and relationships between the metacoupling framework and other concepts, frameworks, and disciplines (e.g. [13,36,56–58]). For example, the metacoupling framework differs from previous frameworks (e.g. [5,55,59–61]) in several ways (Fig. 1). (1) It differentiates human–nature interactions within a system, between adjacent systems, and between distant systems. (2) It explicitly identifies sending, receiving, and spillover systems as well as causes, effects, and agents for each system. (3) It emphasizes both socioeconomic and environmental interactions and feedback through various flows within and between different systems. (4) It integrates not only ecosystem services or nature's contributions to people (e.g. [55,59]) but also negative impacts of nature on humans (e.g. hazards, disasters) [62,63]. (5) It connects with scientific and societal goals such as the UN Sustainable Development Goals [7].

The metacoupling framework is simple enough to be flexible for different contexts and various issues related to humans, nature, and their interactions (Fig. 1). Each component of the framework can be further specified with more detailed subcomponents and sub-subcomponents, etc. For example, the social and economic components include social justice and economic equity [64]. Achieving sustainability in one system at the cost of adjacent or distant systems is a form of injustice across space [65], which has received much less attention than injustice within a system. The framework can also help address related issues such as fairness among different systems [56].

## MAJOR ADVANCES IN SCIENTIFIC DISCOVERIES

### Revealing effects of metacoupling on SDG performance and spatial interactions

Applications of the metacoupling framework have broadened studies on SDGs within borders to SDGs both within and across borders. They are in contrast to previous work that concentrated on SDG performance as well as synergies and trade-offs within a specific place.

**Figure 2.**
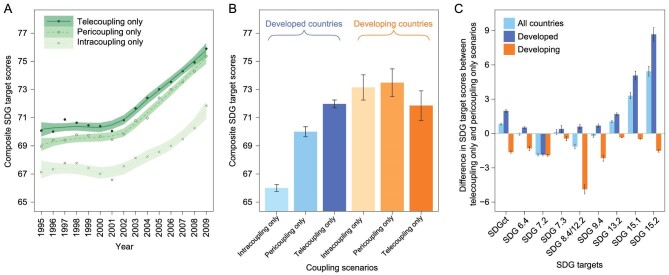
Scores of SDG targets under telecoupling, pericoupling, and intracoupling scenarios. (A) Dynamics of SDGct scores (composite target scores—overall performance in achieving all evaluated SDG targets) for all countries under the three scenarios. (B) SDGct scores for developed and developing countries under each scenario. (C) Differences in SDG target scores between the telecoupling and pericoupling scenarios. The error bars refer to the standard errors in the SDG target scores (n  =  15). Adapted with permission from [8]. Copyright 2020, Nature Portfolio.

Metacoupling has important impacts on progress toward SDGs within and across national borders. For example, evaluating the impacts of global trade during 1995–2009 on nine environment-related SDG targets shows that trade between distant countries (those not sharing land or maritime boundaries, telecoupling) had a higher positive effect on progress toward SDG targets (Fig. 2A) [8] than trade between adjacent countries (those sharing land or maritime boundaries, pericoupling). Countries without trade (intracoupling) had the lowest scores. These effects were true throughout the study period (Fig. 2A). Also, developed countries experienced different impacts of metacoupling than developing countries (Fig. 2B). Furthermore, developed countries had a larger boost from telecoupling than from pericoupling. In contrast, developing countries suffered more from telecoupling than from pericoupling (Fig. 2C) [8].

**Figure 3.**
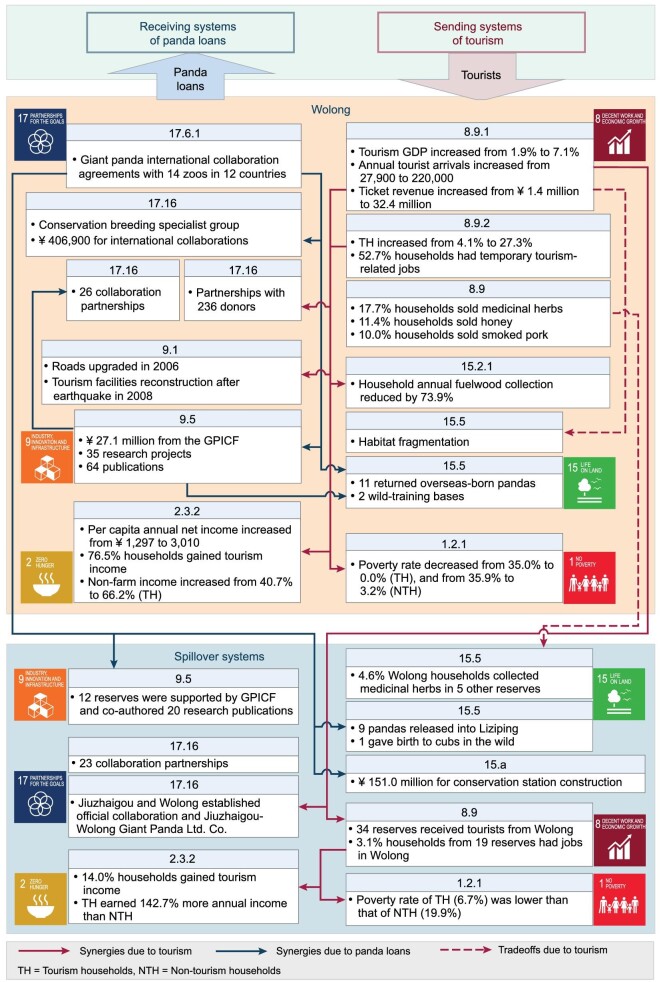
SDG synergies and trade-offs within and across boundaries of Wolong Nature Reserve and other nature reserves for giant panda conservation in China due to tourism and panda loans. Bold numbers refer to specific SDG targets and indicators. GPICF = Giant Panda International Collaboration Fund. Adapted with permission from [10]. Copyright 2020, Elsevier Inc.

Synergies and trade-offs among SDGs also exist within and across system boundaries at international and subnational levels. A synthesis of 22 cases of tourism and wildlife translocations across six continents indicates 33 synergies and 14 trade-offs among 10 SDGs within focal systems (sending or receiving systems of tourists and wildlife) and across systems including spillover systems [9]. A study on the effects of tourism and panda loans (giant pandas from Wolong Nature Reserve in southwestern China loaned to outside zoos) on six SDGs in Wolong and the other 66 panda reserves revealed 17 synergies and two trade-offs [10] (Fig. 3). Among them, there were 10 synergies and one trade-off within Wolong, and seven synergies and one trade-off across reserve boundaries [10].

### Untangling complex interactions among intracoupling, pericoupling, and telecoupling

The metacoupling framework has helped expand studies on human–nature interactions from within a system to within a system as well as between adjacent and distant systems concurrently. Results demonstrate that intracoupling, pericoupling, and telecoupling can have synergistic effects. For instance, telecoupling of information about abundant groundwater in Twin and Chippewa Creek watersheds of Osceola County in the state of Michigan in the US inspired Nestlé (a multinational food- and drink-processing conglomerate corporation with headquarters in Vaud, Switzerland) to build a large-capacity groundwater well near the creeks and draw water (100 000 gallons per day, intracoupling) for a drinking water–bottling facility. Such a facility generated many local effects [66], including 284 jobs and $24.2 million in total economic activity in 2017 alone. Its pericoupled effects include 634 jobs and $71.9 million in total economic activity in 2017 in the five-county region bordering the creeks [66].

Pericoupling and telecoupling amplify intracoupling. Land use (intracoupling) in an upland rural area on the Chinese side (focal system) of the China–Myanmar border was boosted by labor migration from inland Myanmar (telecoupling) and the Myanmar side of the border (pericoupling) [67]. More specifically, the metacoupling framework helped identify flows of the laborers between Myanmar (sending system of the laborers) and China (receiving system of the laborers). Results indicate the cheap laborers from Myanmar are crucial for agricultural intensification in the focal system because of its own aging population and its own young people moving to work in cities [67].

Increases in one type of coupling may reduce other types of coupling [16] and one type of coupling may generate benefits on one scale at the cost of other scales [16]. For instance, food exports may boost regional or national GDP, but may compromise food security at the local level if exports reduce food supply for local consumption without substitutes, causing distributive equity effects [16]. This is the case of the Magallanes region in southern Chile, where wild fish captures and exports expanded after 1980 while the regional consumption of fish was one of the lowest in the country due to a shortage of fish [16]. Such spatial trade-offs generate or increase spatial inequalities [16].

Telecoupling, pericoupling, and intracoupling interact indirectly. Take Paraguay's soybean production and exports as an example [18]. Paraguay does not have direct trade with China, but exports soybeans to neighboring countries such as Brazil and Argentina, and distant countries such as Russia. All these countries importing Paraguay's soybeans export soybeans or soybean products to China. As a result, Paraguay's intracoupled soybean production, and pericoupled and telecoupled soybean exports have increased because they are indirectly affected by China's increasing soybean demand, which has driven more soybean exports from countries such as Brazil and Argentina that in turn import soybeans from Paraguay [18]. In another example, soil conservation through erosion control upstream improves soil fertility and land productivity upstream (intracoupling) [20]. It also influences the water quality and lifespan of reservoirs in the midstream area (pericoupling) and the downstream area as well as water quality and sediment in the coastal areas and oceans (telecoupling) [20]. In this case, intracoupling affects pericoupling, which in turn affects telecoupling. The application of the metacoupling framework to soil erosion-transport-deposition across space helps treat upstream, midstream, and downstream areas as a metacoupled human and natural system, and the framework serves as a platform to integrate supply and demand of soil conservation services across different scales [20].

Multiple types of metacoupling interact positively or negatively. In a study that analyzed the evolution and interactions of multiple global flows of virtual materials (water, energy, land, CO_2_, nitrogen, and financial capital embodied in international trade) among adjacent countries (pericoupling) and among distant countries (telecoupling) from 1995 to 2008, results show that financial capital flows almost doubled, flows of CO_2_ and energy increased ∼60%, water ∼50%, and nitrogen 10%, but land declined 9% [68]. Different types of virtual material flows tended to have synergistic effects, and CO_2_ and nitrogen flows tended to have more powerful synergetic effects than the others [68]. China's interprovincial virtual water-energy networks in 2007 also tended to boost each other [69].

### Unveiling spatiotemporal dynamics and effects of intracoupling, pericoupling, and telecoupling

**Figure 4.**
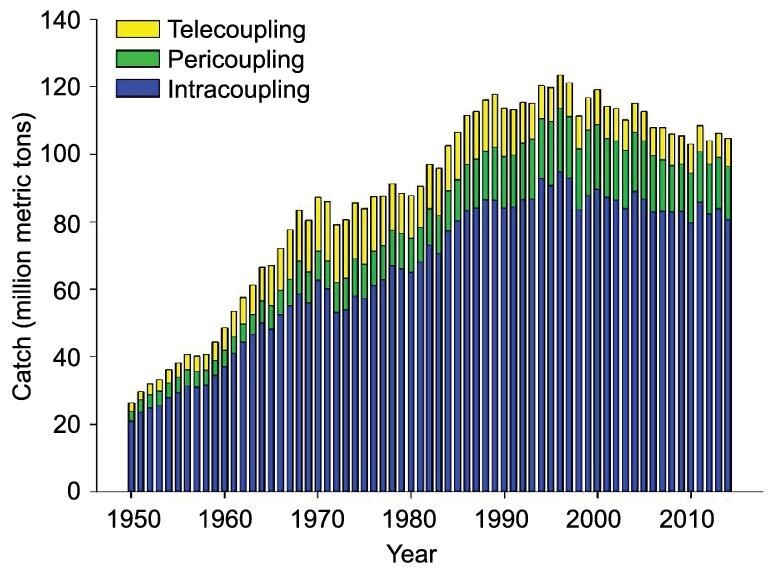
Global metacoupled marine fisheries catches during 1950–2014. Intracoupling refers to industrial, artisanal, subsistence, and recreational catches and intranational flows from fishing within nations’ own exclusive economic zones (EEZs). Pericoupling denotes industrial catches in, and flows from, EEZs of adjacent nations. Telecoupling represents industrial catches in, and flows from, EEZs of distant nations. Adapted with permission from [70]. Copyright 2020, MDPI.

**Figure 5.**
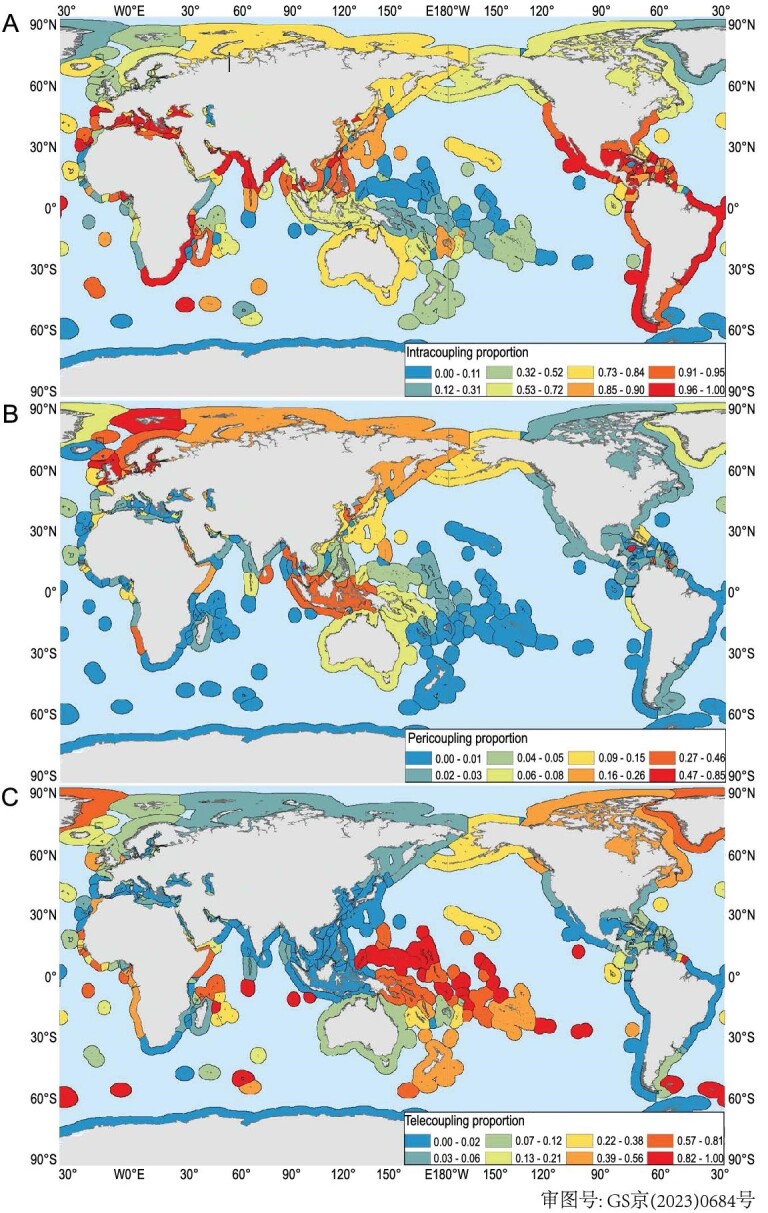
Proportion of marine fisheries catches and flows out of total catches in the exclusive economic zones (EEZs) during 1950–2014. (A) Intracoupling refers to industrial, artisanal, subsistence, and recreational catches and intranational flows from fishing within nations’ own EEZs. (B) Pericoupling denotes industrial catches in, and flows from, EEZs of adjacent nations. (C) Telecoupling represents industrial catches in, and flows from, EEZs of distant nations. Modified with permission from [70]. Copyright 2020, MDPI.

The metacoupling framework has, simultaneously, facilitated the comparisons of dynamics and effects of intracoupling, pericoupling, and telecoupling. Research shows that intracoupling, pericoupling, and telecoupling change over time and across space. For instance, a project quantified global marine fisheries’ catches within nations’ exclusive economic zones (EEZs, intracoupling), within adjacent nations’ EEZs (pericoupling), and within distant nations’ EEZs and high seas (telecoupling) during 1950–2014 [70]. Results show that intracoupling accounted for 73% of all catches (4.3 billion metric tons [MT]), while pericoupling shared 13% (748.9 million MT) and telecoupling 14% (791.7 million MT). In general, all couplings increased considerably from 1950 until the late 1990s, when some declines began (Fig. 4). In some years, one of the coupling types increased or decreased more than others [70]. The three types of coupling interacted differently across fisheries. For example, intracoupling tuna artisanal and subsistence catches declined with increasing pericoupling and telecoupling industrial fishing, respectively. Cod subsistence catches decreased with increasing pericoupling and telecoupling industrial fishing and intracoupling artisanal fishing [70]. In terms of spatial distribution (Fig. 5), intracoupling was prevalent worldwide during 1950–2014, with an average of 208 EEZs (75.4% of all EEZs) annually. Pericoupling had the lowest frequency of occurrence (6.1% of EEZs) but was significant in northern and western European waters, which are close to developed countries. Telecoupling (18.5% of EEZs) was widespread in Oceania and western Africa, where there is high fish productivity but relatively limited fishing infrastructure, governance, and enforcement [70–72].

Temporal and spatial dynamics are also common in other metacouplings. For example, the percentage of China's provinces that transferred more virtual energy than was used within the provinces jumped from 23% in 2007 to 37% in 2012 [73]. The ratios of imported virtual energy to internal energy varied substantially among provinces from ∼0.2 to almost 1.5 in 2007 and from ∼0.1 to 2.6 in 2012. Furthermore, there was a large increase in the percentage of provinces with more distant virtual energy trade than adjacent trade over time (from 73% in 2007 to 100% in 2012). The ratios of distant to adjacent virtual energy trade also differed remarkably among provinces, from ∼0.7 to 4.2 in 2007 and from ∼1.1 to 29.1 in 2012 [73].

Effects of metacoupling differ among sending, receiving, and spillover systems. A study in the globally important soybean-producing state of Mato Grosso in Brazil illustrates such differences [15]. The vast majority of soybeans produced there went to other states in Brazil or other countries (receiving systems of soybeans). In 2018, ∼56% of the soybeans produced in the state were exported to China and the European Union. Soybean production has generated substantial deforestation and economic growth within focal municipalities (sending system of soybeans) in Mato Grosso. Moreover, it has led to pericoupling effects such as deforestation in adjacent municipalities (receiving systems of information from the focal municipalities about soybean production and trade), which did not gain economic benefits until they began to produce soybeans later. Deforestation for soybean production causes CO_2_ emissions and reduces CO_2_ sequestration, contributing to global climate change. Thus, many other countries and regions that do not have soybean trade with the state of Mato Grosso are spillover systems as they are affected by climate change with contributions from soybean production and trade. The results suggest that environmental effects are more widespread than socioeconomic benefits of soybean production and trade [15].

Studying transfers of two or more types of resources simultaneously enables comparison of the gains and losses of different types of resources across metacoupled systems. A study on interprovincial virtual water and energy transfer networks in China in 2007 demonstrates that over 40% of provinces obtained one type of resource (energy or water) but lost the other type of resource (water or energy), and 20% of provinces lost both energy and water [69]. The rest of the provinces gained both energy and water [69]. Approximately 27% and 33% of the provinces depended more on interprovincial trade than on their own energy and water resources, respectively. Furthermore, resource inequality was further enlarged because relatively energy/water-scarce provinces provided ∼40% of transferred energy/water to energy/water-abundant provinces [69].

Effects of metacoupling can be quantified in many ways. Besides common measures such as those mentioned above, a new way is the recently proposed indices of efficiency of intracoupling, pericoupling, and telecoupling [74]. Using the Middle Route of China's South-North Water Transfer Project (the world's largest water transfer project [75]) as an example, the water efficiency of intracoupling was measured based on the ecological benefits, supplemented by socioeconomic benefits, as a result of construction in Danjiangkou Reservoir (a water source and sending system in South China). Ecological benefits include reduced pollution from emissions of chemical oxygen demand (COD, the amount of oxidant consumed in the treatment of organic pollutants) and sewage into the river while socioeconomic benefits encompass value of agricultural products and GDP per capita. The pericoupling efficiency measures the ecological and socioeconomic effects of the water-sending area (Danjiangkou Reservoir) on the downstream area. Ecological effects include COD emissions and reduction in water volume (and risk of floods) due to the water transfer to distant receiving systems. Socioeconomic effects encompass the value of agricultural products, value of industrial products, GDP per capita, and water consumption by residents. The telecoupling efficiency refers to the effects of water transfer from the water-sending area on distant water-receiving areas. Ecological effects include COD emissions and the amount of sewage treatment with the increase in water volume (which also reduces water shortage and improves water quality) in the distant receiving systems. Socioeconomic effects encompass value of agricultural products, value of industrial products, financial investment in water management, GDP per capita, population growth, and water use in the distant receiving areas of North China [74]. The results indicate overall high water efficiency, with telecoupling efficiency being the highest (1.09), followed by pericoupling efficiency (0.82) and intracoupling efficiency (0.61) [74].

### Identifying new network attributes

A number of studies have quantified metacoupled systems as networks. A network consists of nodes that are connected by links (or ties or edges). Correspondingly, a metacoupled system consists of individual coupled human and natural systems (sending, receiving, and spillover systems) that are connected by flows. (Other components such as causes and effects are often not explicitly considered in a network. In social network analysis, agents such as individuals are treated as nodes.) A quantitative network analysis of metacoupled systems through soybean trade among 217 countries during 1986–2013 indicate that the network had 165 distinct cliques, but only a few key disproportionately influential players (Brazil, China, and the US) [76]. The total network density (proportion of actual connections over total possible connections) jumped 5-fold with a progressively lower number of heavy-trade countries, which generated concerns over food security and sustainability. Furthermore, there were close positive associations between cumulative soybean exports and cumulative loss of tropical forests [76]. A network analysis of a community-supported fishery concluded that it is useful to understand emerging food supply chains and enhance fishery management under both normal situations and crises [77]. Results from a study using social network models indicate that global tourism networks of 124 countries became very consolidated from 2000–2013 [78]. Another network analysis of 2133 watersheds (sending systems of ecosystem services) for 317 cities (receiving systems of ecosystem services) worldwide shows that protected wetlands in the watersheds help sustain freshwater provision to cities and forest cover in protected areas of watersheds can enhance the capacity of large dams in lowering sediment loads and generating hydropower [79].

### Uncovering feedbacks across metacoupled systems

Feedbacks are common in metacoupled systems. The use of long time-series data can be particularly helpful to uncover feedbacks, which often take a long time to emerge. For example, a study on global soybean trade among 217 countries during 1986–2013 has revealed a positive feedback between sending and receiving systems: countries with established trade partnerships had a higher likelihood to enlarge trade relationships [76]. In many cases of payments for ecosystem services, payments from distant systems are made for farmers to restore and conserve ecosystem services in various systems (e.g. [80]). As progress for ecosystem services is achieved, more payments are made (e.g. [81]). This is a type of positive feedback. However, if the goal is not achieved, there may be negative feedbacks in which the farmers receive partial payments, no payments, or even punishments [82].

### Detecting and integrating hidden phenomena and overlooked issues

**Figure 6.**
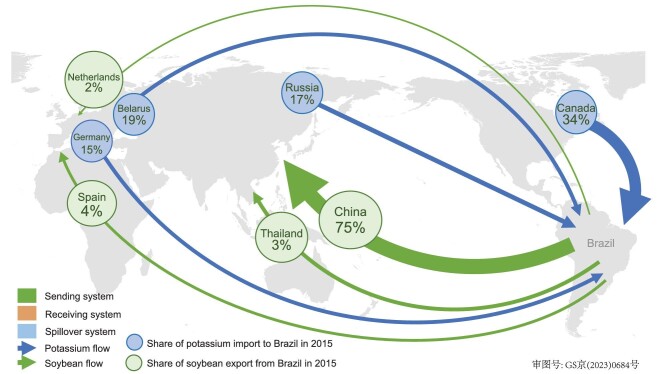
Flows of soybeans from Brazil (sending system) to China and other countries (receiving systems) and flows of fertilizers (potassium) from countries such as Canada and Russia (spillover systems) to Brazil for soybean production. Also shown are the amounts of soybean exports from Brazil to four representative countries in 2005 and 2015, and potassium imports to Brazil from four representative countries in 2005 and 2015. Modified with permission from [56]. Copyright 2018, Elsevier Inc.

Applying the metacoupling framework can help detect hidden phenomena and discover overlooked issues. Spillover systems were traditionally overlooked but are very common. For example, Brazil has been a major sending system of soybeans to many receiving systems such as China and some countries in Europe [56]. To produce soybeans, Brazil largely depends on fertilizer imports from countries such as Russia (Fig. 6) [56]. In other words, countries like Russia providing fertilizers for soybean production are spillover countries of soybean production in Brazil and its resulting trade. The current Russia–Ukraine war could have major negative effects on biodiversity in Brazil because less fertilizer can be exported from Russia, and Brazil is planning to produce more fertilizer by developing Indigenous land rich in biodiversity [83].

Spillover effects (effects on spillover systems) can be much larger than effects between sending and receiving systems. In the case of panda transport from Chengdu in China to Edinburgh Zoo in the UK, the flight of the FedEx aircraft to carry the two pandas actually originated in Memphis, Tennessee, USA [84]. The airplane made a stop in Anchorage, Alaska, to add more fuel before flying to Chengdu airport, the closest airport to Wolong Nature Reserve where the pandas lived. Then the pandas from Wolong were loaded and transported to Edinburgh. The story does not end here as the aircraft then flew back to Memphis. So, Memphis and Anchorage are spillover systems in terms of stop points of the flight, and the rest of the world was the spillover system in terms of CO_2_ emissions. Actually, the distance and the amount of CO_2_ emissions along the Memphis–Anchorage–Chengdu–Edinburgh–Memphis route are several times larger than those from Chengdu to Edinburgh [84]. This example illustrates the importance of identifying hidden spillover systems and spillover effects.

Besides the hidden spillover systems and effects, the framework also helps detect many other issues that are often overlooked. For example, a homogeneous statewide regulation of groundwater withdrawals in the state of Michigan (USA) ignores the differences in intracoupling in different streams and watersheds as well as their pericoupling and telecoupling [66]. As a result, relevant stakeholders and their concerns are neglected. Such negligence causes negative social and ecological outcomes. Using insights from applying the metacoupling framework can identify and improve relationships with relevant stakeholders in groundwater governance and generate positive outcomes across multiple scales [66].

The metacoupling framework is useful to identify the influences of various factors (e.g. internal, adjacent, and distant) on socioeconomic–environmental dynamics, processes, and effects in particular locations. For example, water use in China is simultaneously affected by local factors (e.g. population size) and nonlocal factors (e.g. migration) [85]. Such findings can help develop effective ways to save water in China and many other countries. In China's Loess Plateau region, internal and external factors such as local economy and investment dominated the influences on income of rural households [86], and rural nonfarm employment and rural–urban migration enhanced afforestation effectiveness while grain production and investment in fixed assets reduced afforestation effectiveness [87].

The metacoupling framework can help connect all the issues and components, including those hidden and overlooked. It can, for example, expand the traditional research on ecosystem service flows that largely focused on beneficiaries and supplies (e.g. [88]) by incorporating other components related to ecosystem service flows, such as agents, causes, and effects, as well as spillover systems in addition to sending systems (which include supplies) and receiving systems (which include beneficiaries) [75,89]. A study of the Middle Route of the South-North Water Transfer Project of China identified causes of the project and additional effects such as biological invasion and threats to biodiversity in spillover systems [90]. The framework can also help integrate the effects that may not be quantifiable but can enhance the qualitative understanding of the relationships among different components such as agents and flows [90]. Furthermore, the framework systematically helps incorporate the values and risks from the project, offers a theoretical reference regarding the responsible parties for ecological compensation, and helps evade unnecessary development and improper appropriation of resources [90].

### Expanding the nexus approach

The metacoupling framework has helped to expand the nexus approach across space and across scales. The traditional nexus approach has focused on connections among two or more sectors such as energy, food, and water within a particular place and at a particular scale. From the metacoupling perspective, a sector interacts with not only other sectors within a place (intracoupling), but also all sectors in adjacent places (pericoupling) and distant places (telecoupling) [91]. Incorporating interactions with other places also enables the researcher to bridge multiple scales and examine overlooked drivers and regions in spillover systems [91]. Such an expanded nexus approach has been implemented through quantifying the effects of irrigated agriculture on the food–energy–water–CO_2_ nexus across food sending, receiving, and spillover systems [26]. Results show that the North China Plain (NCP, a food sending system) provided food to, and enhanced food sustainability in, the rest of China (food receiving system) but led to its own water unsustainability (by consuming four times more water than its yearly renewable water), with large differences in the food–energy–water–CO_2_ nexus among counties within the NCP. To provide water for food production in the NCP (and some cities in North China), the South-North Water Transfer Project was constructed [75], occupying much land in Hubei Province (the spillover system), which was rarely part of the food trade [26]. The expanded nexus approach has also been applied to other situations, such as China's interprovincial flows of the energy–water nexus [69] and global flows of virtual CO_2_–energy–land–water–nitrogen–financial capital nexus across national borders [68].

### Re-examining theories

The metacoupling framework is useful for testing theories. As a guiding principle in many disciplines, such as geography and ecology [57], Tobler's First Law of Geography states ‘near things are more related than distant things’ [92]. However, metacoupling analyses indicate that under many situations, empirical evidence is opposite to Tobler's First Law. For example, Brazil and the US, the world's largest soybean producers and exporters, had more soybean trade with distant countries than with adjacent countries among 217 countries during 1986–2013 [76]. A study on six global flows of virtual materials (water, energy, land, CO_2_, nitrogen, and financial capital) during 1995–2008 also shows that telecouplings were much stronger than pericouplings [68]. Another study found that ∼73% and 83% of provinces in China relied more on distant provinces than adjacent ones in terms of total volumes of traded energy and water in 2007, respectively [18,22,69]. A systematic review indicates that the metacoupling framework has a much broader applicability than Tobler's First Law across seven major sustainability topics: agricultural development, conservation, governance, land change, species migration, tourism, and trade [93]. Such findings do not mean that Tobler's First Law is invalid in all situations. In fact, some cases are consistent with the law, such as the negative association between the numbers of international tourists and geographic distances across a total of 124 sending and receiving countries during 2000–2013, suggesting a preference to visit countries nearby [78]. Nevertheless, the results from metacoupling analyses indicate that adjacency is often an inadequate predictor of interactions among systems, and re-examination of all relevant theories in various disciplines (e.g. niche theory, trade theory, scaling theory, and livelihood theory) is warranted from the metacoupling perspective [57].

### Unfolding transformation among noncoupling, coupling, decoupling, recoupling

**Figure 7.**
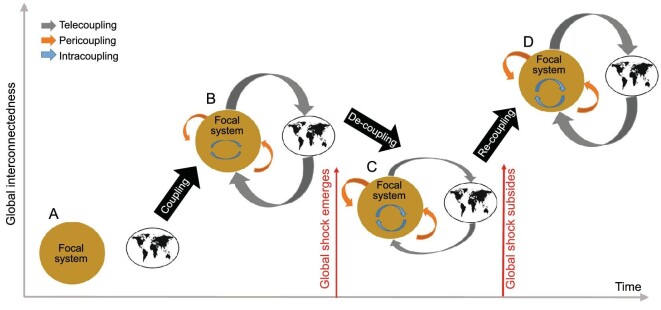
Diagram of hypothetical transformations among noncoupling (A), coupling (B), decoupling (C), and recoupling (D) of intracoupling, pericoupling and telecoupling (e.g. economic development, international trade, tourism, migration) under global shocks (e.g. economic recession, pandemic, war). Arrow thickness indicates relative magnitudes of intracoupling, pericoupling, and telecoupling. Modified with permission from [94]. Copyright 2022, Springer.

Metacoupling is dynamic over time and under influences of many factors. It may experience four stages of transformation: noncoupling, coupling, decoupling, and recoupling (Fig. 7). The stage of noncoupling refers to the status where there are no interactions among different components. The stage of coupling emerges with interactions among different components. Under influences of various factors such as shocks (e.g. economic recession, pandemic, and war), the coupling may become weaker or even dissolve (the stage of decoupling). After factors that cause decoupling to subside or disappear, recoupling may occur (i.e. weakened or dissolved interactions may be recovered). The last three stages were recently identified [94], but it is important to add the first stage (noncoupling), which has important implications for sustainability.

**Figure 8.**
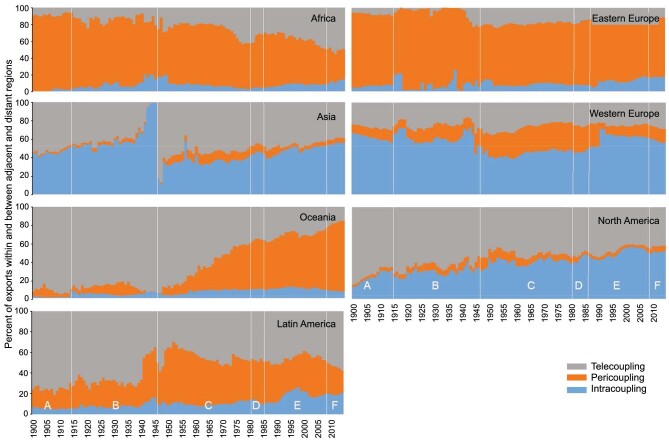
Temporal dynamics of the relative contribution of intracoupling, pericoupling, and telecoupling to metacoupling of world regions (Africa, Asia, eastern Europe, Latin America, North America, Oceania, and Western Europe), represented as the percentage of merchandise exports within a region (intracoupling), between adjacent regions (pericoupling), and between distant regions (telecoupling). For example, exports within Africa are intracoupling, exports between Africa and Western Europe are pericoupling, and exports between Africa and North America are telecoupling. The vertical lines delineate time periods with different global processes (A = Belle Epoque; B = Two World Wars, economic depression and the Spanish flu pandemic; C = Post-war; D = Economic recession of the early 1980s; E = Establishment of the World Trade Organization and growth of e-commerce; F = Great Recession of the late 2000s–early 2010s). Adapted with permission from [94]. Copyright 2022, Springer.

Furthermore, a specific stage of transformation may occur in intracoupling, pericoupling, and telecoupling simultaneously or separately. Fig. 8 shows the four stages in terms of exports within and between adjacent and distant world regions (five continents as well as eastern Europe and western Europe) from 1900 to the early 2010s [94]. Take Africa as an example. For intracoupling, there was noncoupling during early 1900s, coupling emerged around 1905, decoupling occurred around 1950, and recoupling began around 2010. For pericoupling, decoupling dominated the entire period, although recoupling occurred in some years. For telecoupling, the general trend was increased coupling, although decoupling also occurred in some years. Asia was quite a contrast to Africa. Intracoupling accounted for a large portion and pericoupling had the least portion for almost the entire time period, and noncoupling for pericoupling and telecoupling occurred in 1943 and 1944 (just before the end of World War II).

At the subnational level, metacoupling research has also led to the identification of noncoupled regions where humans and nature did not interact within the regions or with other regions. For example, a study showed various relationships between people and ecosystem services within Magallanes and Chilean Antarctica regions of southern Chile (Magallanes region henceforth, intracoupling), between the region and adjacent regions (pericoupling), and between the region and the rest of the world (telecoupling) [16]. The region is particularly important because it encompasses an extensive territory with low population density, has several natural navigable corridors that bridge Asia-Pacific and the rest of the world, and is the entry to the Antarctic continent, thus enhancing the movement of people, information, capital, and technology. The results indicate many intracouplings, pericouplings, and telecouplings. The study also identified noncoupled regions whose ecosystem services are not provided to people within the region or in other regions. Furthermore, for different ecosystem services (sense of place, food from aquaculture, recreation, artisanal fisheries), there are different noncoupled regions [16].

## IMPLICATIONS FOR PROMOTING GLOBAL SUSTAINABILITY

### Achieving SDGs across space worldwide

To achieve SDGs, many suggestions have been offered but are largely fragmented. The metacoupling framework can bring different measures together in a coherent and systematic way. It can help realize SDGs in a specific place as well as adjacent and distant places [12]. Cooperation and coordinated efforts are needed to enhance synergies and reduce trade-offs among SDGs across space. For instance, to eliminate hunger in every country around the world (SDG 2), it is essential to account for human–nature interactions within a country and its relationships with adjacent and distant countries [12]. On one hand, it is important to ensure adequate domestic food production. On the other hand, it is necessary to import food from other countries when domestic production is insufficient to meet the demand or export food to other countries when there is surplus. Food production and trade need to optimize economic efficiency while minimizing environmental impacts such as carbon emissions (SDG 13: Climate Action) during food production, storage, and transport. For instance, many countries with no global biodiversity hotspots export food to countries with global biodiversity hotspots [25], thus reducing impacts of food production on biodiversity (SDG 15: Life on Land). Similarly, the framework is also helpful for achieving other SDGs worldwide. SDG 14 (Life below Water) can be affected by fertilizers and pesticides for food production (SDG 2) in terrestrial systems where runoff flows into water bodies [95]. To achieve SDG 14, it is important to protect oceans but also minimize or eliminate pollutants from adjacent and distant terrestrial systems [12].

### Amplifying benefits of ecosystem restoration across boundaries and across scales

Ecosystem restoration is important for global sustainability because it can prevent, halt, and reverse the degradation of ecosystems [96]. However, traditional ecosystem restoration research and practice usually focused on the place where the restoration occurred, although other places can be affected by or affect the outcomes at the restoration place. Ecosystem restoration influences other places at multiple scales through flows of material, energy, and information [97]. For instance, increased vegetation and the associated carbon sequestration from restoration influence global carbon concentration via atmospheric circulation. The metacoupling framework helps motivate ecosystem restoration to reduce trade-offs and enhance synergies among multiple SDGs at multiple spatial scales [97]. It can aid in providing comprehensive information on the performance of ecosystem restoration by quantitatively understanding the effects of production, living, and ecological functioning under ecosystem restoration in the restoration place, adjacent places, and distant places [98]. Furthermore, the beneficial effects on SDGs at the restoration place should minimize the negative effects on the SDGs in places nearby and far away worldwide [97].

### Augmenting transboundary management

The metacoupling framework is naturally powerful to guide transboundary management because it inherently integrates human–nature interactions within and across boundaries (e.g. regional, national). It has been applied to address management of transboundary watersheds [99,100]. In the Limpopo River basin in southern Africa, a study found the framework valuable in several ways [99]. First, the spatially explicit nature of the framework would encourage the cooperation of multiple agents (stakeholders) throughout the watershed of ∼400 000 km^2^, home to 18.8 million people in South Africa, Botswana, Zimbabwe, and Mozambique, with more than 100 dams and thousands of small reservoirs and irrigation projects [99]. One fundamental cooperation is to collect and share comparable data for guiding water management decisions and enhancing cooperative management. Second, the framework provides a conceptual foundation for analyzing local management practices within the watershed as well as regional, national, and international relationships that influence management decisions. The decisions made at the local level are both affected by and can affect water quantity and quality in other parts of the basin and beyond. Thus, it is essential that a watershed management plan address human–nature interactions across scales, including agents, flows, causes, and effects. The framework provides a means to treat and examine all socioeconomic and ecological components across all scales together for effective decision making. Third, the framework can help a variety of stakeholders, such as managers in different water districts, governments from local to regional to national levels, nongovernment organizations, irrigation association committees, and national parks to work collectively toward sustainable use of the entire watershed. Fourth, the framework differs from other frameworks for watershed management in its ability to integrate human–nature interactions within as well as between adjacent and distant systems [99].

### Broadening spatial planning

Spatial planning is important to shape the distribution of people and human activities. Traditional spatial planning largely focused on conditions in a specific geographic area with inadequate consideration of interactions with adjacent and distant places, thus creating many unintended negative consequences. Results from metacoupling studies can help spatial planning at different scales to evade negative effects and enhance positive effects on sustainability in several ways [16]. First, they can increase stakeholder awareness and engagement in spatial planning with explicit attention to interactions between the specific area being planned and other areas nearby and far away. For instance, information on the flows between sending and receiving systems can identify the dependence of receiving systems on sending systems as well as impacts on spillover systems. Second, it can protect sending systems such as ecosystem service provisioning areas by reducing the loss of important ecosystem services, constructing plans to restore deteriorated ecosystem services, and tracking how and to whom a specific sending system provides a specific kind of ecosystem service. Third, information about noncoupling—the spatial mismatch between sending and receiving systems—can promote access mechanisms from the perspective of spatial equality and justice [101]. Fourth, the results can generate new incentives (e.g. beneficiary pay-based mechanisms) and investments (e.g. adequate facilities to promote coupling). Fifth, the results can expand the scope of marine spatial planning by including land–sea interfaces and coastal–marine linkages because human activities such as land use in terrestrial and coastal areas affect marine ecosystem service supply [16].

### Boosting supply chains

Metacoupling-based network analysis offers a useful, systematic approach to understand and boost supply chains. It enhances robust community-supported fishery management under normal logistical and financial challenges and extraordinary situations due to crises such as COVID-19 and other emergencies [77]. For example, a metacoupling lens is illuminating to conceptualize, and crucial to manage, supply chains of Fishadelphia (a community-supported program providing access to fresh seafood in Philadelphia, USA) for more effective customer services and stakeholder engagement across different locations [77]. The metacoupling-based network analysis systematically evaluates the systems, flows, agents, causes, and effects of human–nature interactions in fisheries across multiple scales. It is critical to bridge knowledge gaps generated by separate assessments of dynamics in human or natural components within individual fisheries [77]. It is also flexible for different fisheries throughout the world to enhance resilience and adaptive capacity through restructuring and management for socioeconomic and environmental sustainability.

Metacoupling may increase or decrease resilience depending on the composition of intracoupling, pericoupling, and telecoupling. A study indicates that metacouplings regulate the network resilience of Fishadelphia [77]. Specifically, Fishadelphia includes seafood distribution programs or supply chains that provide consumers fresh finfish and shellfish from suppliers (e.g. harvesters, processors). Metacouplings encompass the flows of seafood among stakeholders within subregions in a particular state (intracoupling), between subregions in a state (pericoupling), and between states (telecoupling). The dynamics of metacouplings provided insights for network rewiring (e.g. changes in network structure) after a three-month closure in 2020 due to COVID-19. While intracoupling enhanced Fishadelphia's delivery efficiency and customer safety and satisfaction, pericoupling and telecoupling promoted species diversity and flow consistency before the closure. Before the closure, there was more telecoupling and pericoupling than intracoupling. After the closure, there was more intracoupling relative to pericoupling and telecoupling and a reduction in the species along the supply-side seafood flows [77]. In response to COVID-19, a new delivery program was developed that enabled volunteers to keep coolers at their homes to offer efficient and convenient seafood delivery to customers in distributed locations where customers arrived at different times and maintained social distancing [77]. Consequently, the program minimized COVID-19 transmission while increasing the number of customers, diversified and dispersed the demand-side seafood distribution, and enhanced network resilience [77].

### Empowering small agents in the large world

The vast majority of people in the world are small agents whose actions have small influences individually. Approximately half of the world's poor population in developing nations are smallholder farmers who depend primarily on small, rural land parcels for their livelihoods [102]. They are among small agents who are not shakers and movers like government officials or corporation leaders, yet they are massive in numbers and crucial for meeting global SDGs such as SDG 1 (No Poverty) and SDG 2 (Zero Hunger) [7]. They are parts of the metacoupled world and affected by metacoupling processes such as globalization and global environmental change, including climate change.

A synthesis of 12 cases of smallholder systems worldwide shows they interact with pericoupled and telecoupled systems through flows of goods, information, people, and/or resources [102]. Results also indicate that smallholders intertwined in pericoupled systems (e.g. selling agricultural products to local and regional markets) usually possess strong agency (capability of influencing the formation or operation of flows), which is related more with positive effects (e.g. obtain income from selling agricultural products to local and regional markets) than negative effects. In contrast, smallholders with low agency often suffered negative spillover effects (e.g. in telecoupled systems with external, large investments in agriculture for international exports that made smallholders victims of competition over water and land consolidation for large-scale monoculture farming). These findings suggest increasing agency enables smallholders to improve well-being and achieve SDGs [102]. The synthesis also demonstrates that, in contrast to the traditional place-based approach with a focus on the local dynamics or the prevailing approach of assessing smallholders in the context of globalization that often considered smallholders passively receiving external influences, the metacoupling framework is a holistic approach that incorporates both the smallholder system and the bidirectional flows between the smallholder system and other systems at the village, district, regional, national, and global levels. It also provides insights into the agency of smallholders and pathways to enhance socioeconomic and environmental sustainability [102].

### Shifting from place-based to flow-based governance

The metacoupling framework has laid a foundation for paradigm shifts in the governance for global sustainability from focus on specific places independently (place-based) to flows (movements of people, information, energy, matter, organisms, goods, capital, etc.) among places together (flow-based). Traditional governance largely occurred within political or administrative boundaries and considered different issues separately even though the world has become increasingly metacoupled. As a result, the traditional governance approach often reduced one problem while exacerbating others and solved problems in one place at the cost of other places [91,103]. Flow-based governance stresses that action in one place must account for its relationships with other places both nearby and far away that are linked with various flows. However, there are many challenges to do so (e.g. different interests in different places and higher transaction costs) [104,105]. Thus, it is necessary to consider various factors, such as geopolitics, international and interregional agreements, characteristics of different places [48], institutions [49], historical factors including colonialism [106], and their roles in socioeconomic and environmental relationships across different places. The metacoupling framework is valuable to incorporate various factors and generate new knowledge essential for effective policy making and governance [91]. It can promote changes in governance from focusing on one place to including all relevant adjacent and distant places and from focusing on one issue at a time to considering multiple issues (e.g. biodiversity, food, energy, water, climate) simultaneously and systematically [13]. The flow-based governance also explicitly addresses spatial justice and equity by incorporating flows and effects among different types of systems across space [65].

## FUTURE PERSPECTIVES

Despite the scientific advances and practical implications of applying the metacoupling framework, there are many knowledge gaps and more effective tools and resources are needed to further operationalize the framework. To realize the full potential of the framework in advancing sustainability science (and related emerging fields such as sustainability ecology, sustainability economics, and sustainability geography) and tackling global sustainability challenges, several perspectives are presented below.

### Explore cascading interactions

Research on metacoupling so far has largely concentrated on expanding concepts in various disciplines, such as trade, migration, and water transfer. However, little work has been done on metacoupling as a result of cascading interactions across space, scales, and domains. Cascading interactions refer to a series of processes that result from an event (e.g. climate change, natural disaster, war, economic development, and consumption) in one place cascading and spreading its impacts on similar or different components in other places both nearby and far away through multiple steps. The concept of cascading interactions builds on, expands, and integrates some related disciplinary concepts such as trophic cascades in ecology [107], source-sink dynamics in population biology [108], and globalization in economic geography [109]. For example, large-scale infrastructure projects (e.g. highways, hydropower plants, pipelines, ports, and railways) can affect not only the regions with the projects but also many other related regions, including spillover systems [110]. Cascading interactions are increasingly common and powerful with rapid increases in global environmental change, globalization, conflicts, economic development, human population size, and even faster growth in household numbers [111] due to factors such as divorce [112].

**Figure 9.**
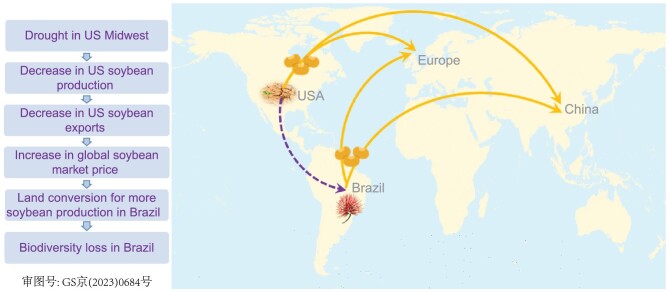
A hypothetical example illustrating cascading human–nature interactions across space. Both USA and Brazil are major soybean producers and exporters for China and many countries in Europe. When a drought occurred in the US Midwest, which is the major soybean production region, it led to a reduction in US soybean production. The reduced production might have lowered soybean exports and boosted soybean price. The price rise could have encouraged farmers in Brazil to convert more forests and grasslands for soybean production. Land conversion causes habitat loss and biodiversity loss. Credit (icons of soybeans, drought, and flower): flaticon (https://www.flaticon.com/free-icon/soybean_5601549); discovermagazine (https://www.discovermagazine.com/environment/what-are-flash-droughts); and Calliandra (https://www.calliandragastronomia.com.br).

As a hypothetical example, Fig. 9 illustrates cascading effects of drought in the US on biodiversity in Brazil, which is more than 7000 km away. Both the US and Brazil are major soybean producers and exporters to China and many countries in Europe [76,113]. The 2012 drought in the US Midwest, the country's major soybean production region, led to reduced production [114,115]. The reduced production might have reduced soybean exports and might have increased soybean price. The price increase could have prompted Brazil to convert more forests and grasslands to soybean production, which causes habitat and biodiversity loss.

Research on cascading interactions goes beyond traditional research about impacts of an event, which often focused on the areas where the events occurred. For example, research on the impacts of natural disasters on biodiversity usually concentrated on areas where the disasters occurred [116]. The same is true for traditional research regarding the impact of war on biodiversity in war zones [117]. The metacoupling framework is conducive to expand research regarding war impact far beyond war zones [83].

### Trace flows across scales and space

Flows are a key component of the metacoupling framework because they link different components and various places. To understand the dynamics as well as mechanisms and effects of flows, data on flows are crucial for several reasons. First, they can be used to measure the strength of the metacoupling. Second, they provide a foundation to understand the mechanisms of flows. Third, large flows often lead to large effects (although the relationships may not be linear and there may be tipping points or thresholds). Fourth, the information from analyzing flow data can help assess important issues such as food security (e.g. whether flows are large enough to meet various demands).

More and complementary types of flow data are needed. For example, regarding trade data, there are many aggregate data at the national and international levels. There are also local data from surveys and interviews with households and individuals (e.g. [118]). Some databases such as Trase [119] connect national data to regional production or consumption, but these are mostly data from modeling. Data that link local to international levels are severely lacking. For instance, there has been an abundance of local data about producers and consumers separately [120]. For most products, there are supply-chain actors (agents) that link producers and consumers, especially those far away. Few data exist regarding how supply-chain actors interact with each other and with producers and consumers systematically, and how feedbacks form and propagate across different places. One possible way to fill this data gap is through the Internet of Things [121], GPS (Global Positioning System) sensors, and artificial intelligence [6].

### Uplift the rigor of causal attribution

Rigorous causal attribution is important to accurately disentangle the complexity of metacouplings and construct informed strategies for effective management and governance of metacoupled systems. However, it is not easy to rigorously identify causal relationships in metacoupled systems, because establishing causal attribution is not straightforward [122]. So far, most metacoupled system studies have used methods such as correlation analyses, mixed-effects models, and gravity models.

Few studies on metacoupled systems have identified causal relationships. For example, using a mixed-effect model and a gravity model, a study identified factors influencing dynamics of international tourist flows among 124 countries during 2000–2013 [78]. A variety of factors exist in sending countries (e.g. GDP per capita, population size), receiving countries (visa-free policies, political stability, violence, terrorism), and sending and receiving countries (e.g. price-level difference, language, geographic distance, number of direct flights between them). Lower transaction costs (e.g. distance, language, and visa policies) were more influential for international tourists than cultural and natural attractions [78]. Furthermore, international tourism was resilient to political instability and terrorism risks. The result of the mixed-effect model may suggest a causal relationship between the factors and dynamics in global tourism networks [78].

Using an augmented gravity model, another study identified the drivers (causes) of China's interprovincial virtual energy and water transfers, such as GDP per capita, precipitation, cropland area per capita of both the sending and receiving provinces, distances between provinces, population sizes, and percentage of industrial GDP in total GDP in the receiving provinces [69]. In the future, there is a great need to boost the rigor of metacoupling causal attribution through triangulating (validating) and deepening evidence for causal mechanisms via integrating qualitative and quantitative methods such as counterfactual analysis and process tracing [122]. Moreover, causal relationships can be further confirmed by systematic assessments of other framework components such as flows, agents, and effects across sending, receiving, and spillover countries [123].

### Enlarge toolboxes

There are many existing tools for metacoupling analysis. Examples include material flow analysis to analyze various material flows [124], agent-based models (ABMs) to analyze agent behaviors and system dynamics [125], causal inference to analyze causal relationships [122], multiregional input-output analysis to determine interdependencies between regions [68], remote sensing to study large-scale system structure and composition [126], footprint analysis [127] and life-cycle analysis [128] to study effects, and network analysis to analyze metacoupled systems as networks [76].

Further applications of the metacoupling framework need to expand a portfolio of tools such as artificial intelligence and machine learning that gather data and enhance models to integrate various data across different places. An emerging approach is to develop digital twins [129], which are digital representations of real-world physical systems or processes. Computer simulation models are examples of digital twins. Although no models can be 100% identical as the real world, many models are useful and can be continuously improved toward the ‘identical twin’ status. One type of useful models is the ABM that simulates interactions among agents and between agents and the environment [130]. Traditional ABMs focus on a specific place [13,131,132]. Recent efforts have led to a telecoupled ABM (TeleABM) that operationalizes the telecoupling framework [125,133,134]. A Telecoupling Toolbox, which includes a suite of spatially explicit tools and can link with different models such as TeleABM, has been constructed to explore the dynamics of telecoupled systems [135,136]. It integrates all components of the telecoupling framework. The Toolbox includes some new tools and integrates many existing methods. It can be used to understand, model, simulate, and predict various components of telecouplings. Besides research, it can also be used to communicate with stakeholders for planning, evaluation, and governance. By building on and expanding TeleABMs and the Telecoupling Toolbox, metacoupled ABMs (MetaABMs) and a Metacoupling Toolbox can be developed to understand metacoupled systems [13].

### Innovate policy

The metacoupling framework can promote policy innovation. More specifically, it can identify gaps in knowledge and action by comparing what is known and done with the components of the framework and their interrelationships. Such gaps can serve as entry points for revising and developing policies regarding new research and action. For instance, as spillover systems are often overlooked, there is relatively little information or action to reduce unintended negative spillover effects [56]. Another example is balance among intracoupling, pericoupling, and telecoupling, which is discussed below.

Achieving an appropriate balance among intracoupling, pericoupling, and telecoupling affects sustainability locally and globally. For instance, food from distant places consumes more energy to transport and thus emits more CO_2_ [137]. However, there are many other costs of food production. If a local place has no suitable weather and other resources such as arable soil and water, producing local food may cost more energy and emit more CO_2_. As a result, the total costs may be higher for locally produced products such as food even after taking the transport costs into account. Localism (belief in local self-sufficiency of resources such as local food) gained more traction after many supply chains were disrupted due to COVID-19 [138], but risks under localism may also be particularly high amid emergencies, such as natural disasters, when the local system depends on support from other places.

While pericoupling and telecoupling can help sustain a local system with high resilience, they also generate risks. Diversifying the sending and receiving systems can reduce the risk of dependency on a single or few systems. Also, from the perspective of global sustainability, it is crucial to detect and reduce negative effects on spillover systems. Further metacoupling research is needed to systematically explore the pros, cons, risks, and pathways.

The balance among intracoupling, pericoupling, and telecoupling is needed at all levels. Usually at the lower level when the system is small, flows between the focal system and other systems would be more important. At the national level, China's ‘dual circulation’ policy is a case that can benefit from the lens of metacoupling. While the dual circulation policy emphasizes economic development, reconceptualizing China's economic development as human–nature interactions across metacoupled systems is essential for sustainability in China and the rest of the world. Using the metacoupling framework could reframe China's internal circulation as intracoupling [22] and the external circulation as intercoupling (i.e. pericoupling and telecoupling). China does not specify the relative importance of external circulation with adjacent countries (pericoupling) vs. distant countries (telecoupling). However, in general, telecoupling can have more drastic impacts because distant places often have more different socioeconomic and/or environmental conditions and can provide different types of flows.

### Elevate financial and human resources

There have been increases in human capacity to address metacoupling and global challenges. For instance, the International Network of Research on Coupled Human and Natural Systems [139] was established in 2008 to facilitate collaborations and communications among researchers in different disciplines. Because young generations of researchers are key for future sustainability efforts, in 1998 with financial support from NASA and Michigan State University (MSU), the NASA-MSU Professional Program was established to support students and junior scholars to attend professional meetings and interact with leading scientists [140]. So far, the program has supported more than 440 students and junior scholars from ∼170 institutions worldwide. The European Commission has supported a PhD program in telecoupling with 15 doctoral students across nine countries [141]. However, given the magnitude and extensive scope of global challenges, many more interdisciplinary researchers are needed.

Some funding agencies such as the US National Science Foundation have provided funding for metacoupling research. To accelerate metacoupling research, there is a strong need for more financial resources from funding organizations in various countries. Furthermore, because metacoupling research needs to consider human–nature interactions across different places, extra financial support is needed for coordination among the relevant places.

## CONCLUDING REMARKS

This article illustrates various functions of the metacoupling framework for scientific discoveries to advance sustainability science and the creation of effective solutions to address global sustainability challenges (Table 1). Fundamentally, the framework is a foundational tool for integrating and understanding human–nature interactions, synergies, and trade-offs across space and multiple scales for global sustainability. It is also a useful platform for researchers and other stakeholders to map out policy interventions and sustainability pathways. The results have enormous implications for local, regional, national, and global efforts such as the UN Sustainable Development Goals, UN Decades on Ecosystem Restoration, Post-2020 Global Biodiversity Framework, and Paris Climate Agreement. The framework provides a good roadmap to realize human–nature harmony everywhere, such as ecological civilization and the 2050 Vision ‘[Humans] Living in Harmony with Nature’ of the Convention on Biological Diversity [142]. It has inspired rethinking for new and more effective policies to minimize negative and enhance positive impacts on sustainability around the world.

The framework can play even more important roles in a wide range of fundamental and applied issues, such as spatialization of sustainability science, creation of a unified theory, and global sustainable development. Despite some concerns over deglobalization, numerous processes and events, such as climate change, war, disease spreads, and disasters, have become more frequent and led to cascading interactions. They will increase the imbalance between supply and demand across space and over time with continuing population growth and even faster household proliferation, thus altering the types and intensity of metacoupling. To realize the full potential of the framework, more financial and human resources for more systematic and integrated efforts are needed to accelerate the further applications of the framework worldwide. Mainstreaming the metacoupling knowledge into decision making and governance can meet the consumption demands of a more populous world while achieving global sustainability.
